# Antitumor activity of Z15-0-2, a bispecific nanobody targeting PD-1 and CTLA-4

**DOI:** 10.1038/s41388-024-03066-5

**Published:** 2024-05-28

**Authors:** Jianyao Zeng, Yuan Fang, Zixuan Zhang, Zhenzhen Lv, Xiaodie Wang, Qian Huang, Zhidan Tian, Jiaguo Li, Wenfeng Xu, Weimin Zhu, Jing Yu, Tao Liu, Qijun Qian

**Affiliations:** 1 School of Medicine, Shanghai University, Shanghai, 200444 China; 2 Shanghai Cell Therapy Group Co., Ltd, Shanghai, 201805 China; 3 Shanghai Mengchao Cancer Hospital, Shanghai University, Shanghai, 201805 China

**Keywords:** Immunotherapy, Cancer immunotherapy, Cancer microenvironment

## Abstract

The combination of programmed cell death protein 1 (PD-1) and cytotoxic T lymphocyte antigen 4 (CTLA-4) antibodies has potential for enhancing clinical efficacy. We described the development and antitumor activity of Z15-0, a bispecific nanobody targeting both the PD-1 and CTLA-4 pathways simultaneously. We designed and optimized the mRNA sequence encoding Z15-0, referred to as Z15-0-2 and through a series of in vitro and in vivo experiments, we established that the optimized Z15-0-2 mRNA sequence significantly increased the expression of the bispecific nanobody. Administration of Z15-0-2 mRNA to tumor-bearing mice led to greater inhibition of tumor growth compared to controls. In aggregate, we introduced a novel bispecific nanobody and have re-engineered it to boost expression of mRNA, representing a new drug development paradigm.

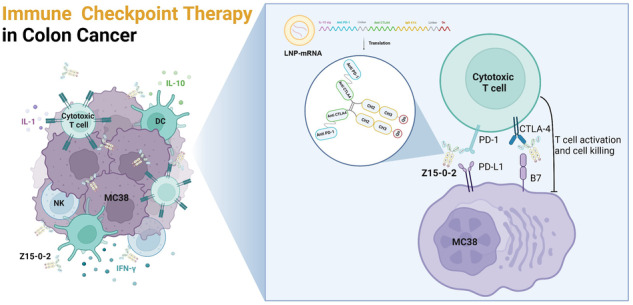

## Introduction

The emergence of immune checkpoint inhibitors often referred to as immune checkpoint blockade (ICI or ICB), represents a significant breakthrough in the field of immune oncology. Both PD-1/PD-L1 and CTLA-4 inhibitors have demonstrated remarkable therapeutic efficacy in treating various cancers.

T cells infiltrating tumors can be suppressed by coinhibitory signals of PD-1 and CTLA-4 [1]. In clinical trials in metastatic melanoma for example, the combination of anti-PD-1 and anti-CTLA-4 has demonstrated the potential for enhancing response rates by up to 60% [2, 3]. However, this treatment is often accompanied with significant side effects, making it challenging for some patients to tolerate the therapy [4].

The occurrence of immune-related adverse events (irAEs) associated with the use of ICIs has been correlated with immune cells carrying fragment crystallizable (Fc) receptors [5]. AK104 (Cadonilimab) a symmetric tetravalent bispecific antibody featuring a Fc null configuration, has received approval from the National Medical Products Administration (China) for treating advanced cervical cancer [6, 7]. In a clinical trial focusing on advanced gastric or gastroesophageal junction adenocarcinoma (NCT03852251), patients receiving AK104 in combination with chemotherapy demonstrated an impressive overall response rate (ORR) of 65.9% [8]. As of writing this in early 2024, AK104 is used in 85 registered clinical trials, including 8 phase III trials. MEDI5752 (Volrustomig), a bispecific monovalent antibody developed by AstraZeneca, targets PD-1/ CTLA-4 and is composed of Tremelimumab (anti-CTLA-4) and an anti-PD-1 monoclonal antibody [9]. Currently, MEDI5752 is undergoing multiple clinical trials, encompassing 3 phase III clinical trials in a variety of tumor types. Other dual-targeting molecules such as QL1706 and KN-46 are included in other studies [10]. However, despite these achievements, antibody-based therapies still face challenges including uneven distribution in tumors, a prolonged serum half-life, and immunogenicity [11].

Nanobodies, also known as a microscale single-domain antibody (VHH), have been found to possess many advantages in comparison to classical immunoglobulin gamma (IgG) [12–14]. It combines the positive characteristics of small molecule antibodies and monoclonal antibodies, including small size, high stability, strong antigen-binding affinity, good water solubility, and natural origin. These attributes make nanobodies an appealing reagent for the development of innovative therapeutic strategies [11]. Caplacizumab (ALX-0681), the first nanobody approved by European Medicines Authority (EMA) and the US Food and Drug Administration (FDA), is a bivalent nanobody used for the treatment of thrombotic thrombocytopenic purpura (TTP) [11, 15].

Stadler et al. conducted a study illustrating that utilization of in vitro-transcribed, pharmacologically optimized mRNA can effectively address the limitations of bispecific T cell-engaging antibodies, thereby facilitating sustained endogenous synthesis of antibodies [16]. In vivo administration typically necessitates the formulation of mRNA into nanoparticles to safeguard against RNase-mediated degradation [17, 18]. Currently, lipid nanoparticles (LNPs) stand as the most advanced and widely used mRNA delivery formulation [19–21]. Moreover, antibody efficacy is intricately linked to mRNA expression levels. The recent identified Exin21 (CAACCGCGGTTCGCGGCCGCT) cis-regulatory motif encoding Qα (QPRFAAA), positioned between the luciferase reporter gene and SARS-CoV-2 envelope (E) protein-coding sequence has potential to enhance protein expression and secretion by improving mRNA stability [22].

In this study, we have developed a bispecific nanobody named Z15-0, with precise targeting capabilities towards PD-1 and CTLA-4. Subsequent comprehensive experiments have been undertaken to demonstrate its biological activity and function in vitro. Moreover, through the optimization of mRNA sequences encoding Z15-0, we have accomplished enhanced expression of Z15-0-2 both in vivo and in vitro. This notable enhancement has been achieved employing the LNP delivery system (abbreviated as LNP-mRNA). As a result, Z15-0-2 has demonstrated improved antitumor activity in our models.

## Results

### The bispecific nanobody Z15-0 exhibits binding affinity towards PD-1 and CTLA-4

**Fig. 1:**
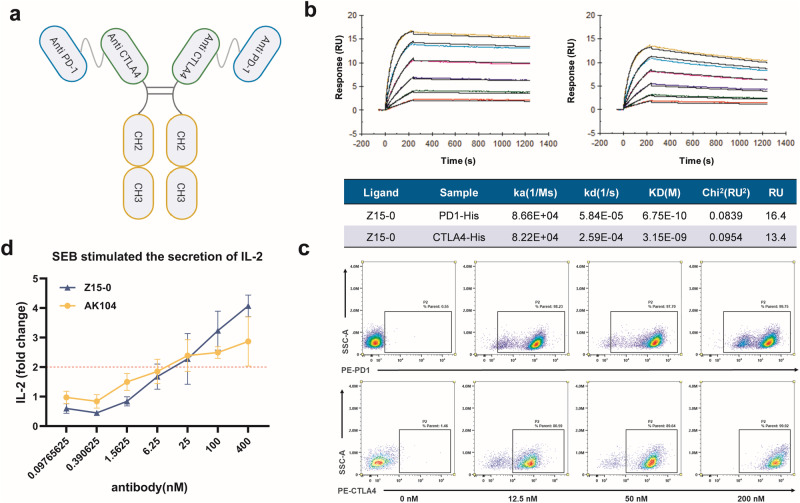
The structure and properties of Z15-0 nanobody. **a** The structure of the Z15-0 nanobody. **b** The affinity of the Z15-0 nanobody was assessed through SPR. **c** The binding ability of the Z15-0 nanobody to PD-1 and CTLA-4 on the cell surface is investigated at different concentrations (0, 12.5, 50, and 200 nM) using flow cytometry. **d** Human peripheral blood mononuclear cells (PBMCs) were stimulated with 500 ng/mL of staphylococcal enterotoxin B (SEB) for 96 h at various concentrations of the Z15-0 nanobody. The secretion of IL-2 was measured using ELISA, and the IL-2-fold change was calculated by dividing the concentration of IL-2 in each experimental group by the concentration of IL-2 at 0 nM antibody concentration. The mean values of duplicate wells are represented by each data point, and the ± SEM is represented by error bars, *n* = 3.

We have developed a new construct, Z15-0, by linking PD-1 and CTLA-4 nanobodies derived from alpacas, using a G4S linker (CN202310338674.1). To enhance its stability and half-life, we incorporated an IgG4 Fc while simultaneously reducing its immunogenicity. This nanobody was screened by VHH MAb (Shcell, China). Figure 1a illustrates the structure of the bispecific nanobody Z15-0. We used SPR to determine its binding affinity towards anti-PD-1 and anti-CTLA-4, resulting in K_D_ of 675 pM and 3150 pM, respectively (Fig.1b).

The binding ability of Z15-0 to PD-1 and CTLA-4 on cell surface were assessed by flow cytometry. Various concentrations of Z15-0 were introduced to these cells and its interaction with the cell surface was detected using an Anti-Camelid VHH Cocktail (PE). As shown in Fig. 1c, Z15-0 demonstrated specific binding interactions with PD-1 and CTLA-4 on the cell surface at different antibody concentrations (200, 50, and 12.5 nM).

Staphylococcal enterotoxin B (SEB) activates T cells and induces IL-2 expression by cross-linking the major histocompatibility complex class II (MHC II) on antigen-presenting cells (APC) with the T cell receptor (TCR) on T cells [23–25]. PBMCs were stimulated with 500 ng/mL of SEB and various concentrations of antibodies. Followed by assessment of IL-2 concentration in the culture medium’s supernatant after 96 hours. As shown in Fig. 1d, Z15-0 significantly increased the secretion of IL-2 by SEB-stimulated PBMC, and this increase was dependent on antibody concentration.

### The presence of Qα insertion does not impede recognition ability towards Z15-0-2

**Fig. 2:**
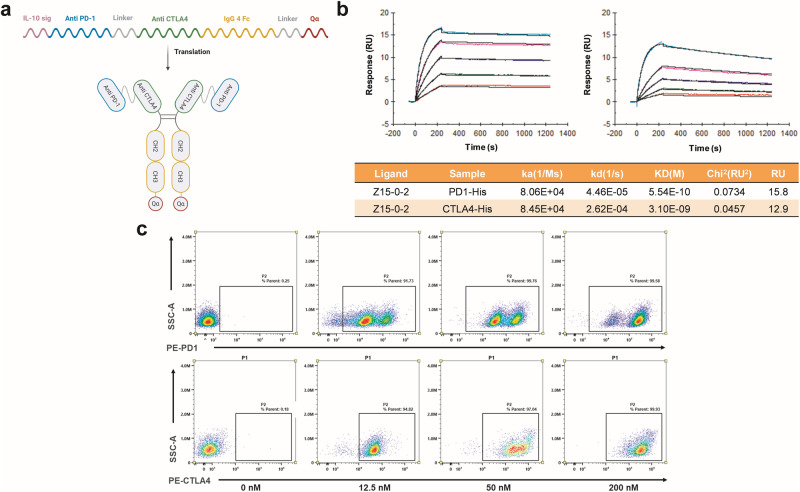
The structure and properties of Z15-0-2 antibody. **a** Z15-0-2 mRNA and antibody structure. **b** The affinity of the Z15-0-2 nanobody was assessed through SPR. **c** The binding ability of the Z15-0-2 nanobody to PD-1 and CTLA-4 on the cell surface was investigated at different concentrations (0, 12.5, 50, and 200 nM) using flow cytometry.

We incorporated Exin21 into the Z15-0 sequence, resulting in the formation of Z15-0-2 (CN202311358648.1). Figure 2a illustrates the mRNA structure of Z15-0-2 and the arrangement of the bispecific nanobodies. The inclusion of the Exin21 sequence, encoding a heptapeptide Qα, is known to boost protein synthesis by improving mRNA stability [22].

The K_D_ for anti-PD-1 and anti-CTLA-4 were determined to be 554 pM and 3100 pM (Fig. 2b) respectively, which exhibited comparable values to those of Z15-0. Moreover, the flow cytometry assay analysis in Fig. 2c demonstrated the binding affinity of Z15-0-2 towards PD-1 and CTLA-4 expressing cells. These findings indicate that the inclusion of Qα does not alter the binding capacity of Z15-0-2.

### Both Z15-0 and Z15-0-2 demonstrate effective inhibition of mouse colon cancer tumor growth

**Fig. 3:**
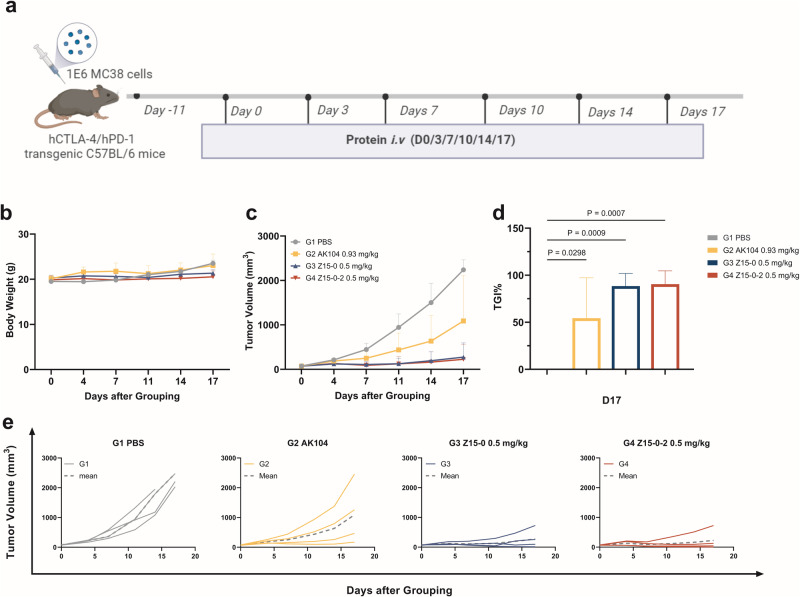
Z15-0 and Z15-0-2 exhibit comparable antitumor efficacy in the hCTLA-4/hPD-1 transgenic C57BL/6 mice colon cancer model. **a** A schematic representation of the experimental design employed to evaluate the in vivo antitumor potential of Z15-0 and Z15-0-2. **b** Alterations in the body weight of the mice were monitored. **c** The tumor volume of the mice was assessed and presented as representative data. **d** The G1-PBS group served as the control, and the tumor growth inhibition (TGI) was evaluated on D17 across the various groups, with TGI% calculated as (1-T/C) ×100%. Statistical analysis was performed using one-way ANOVA, and *n* = 4. **e** Individual tumor volumes were recorded and analyzed.

To assess the effectiveness of Z15-0 and Z15-0-2 bispecific nanobodies and evaluate potential impact of their modification on tumor inhibition, we established an MC38 in vivo tumor model in C57BL/6 transgenic mice. Here, we observed a consistent increase in tumor volume in the PBS control group, confirming the successful establishment of a subcutaneous transplanted tumor model using the MC38 colon carcinoma cell line. The dosing regimens are illustrated in Fig. 3a. On day 17, both Z15-0 and Z15-0-2 at a dosage of 0.5 mg/kg exhibited potent TGI% values of 88.50% and 90.52%, respectively, significantly outperforming those of the control group. In comparison to AK104 (54.28%), both bispecific nanobodies demonstrated superior TGI%, indicating enhanced tumor growth suppression (Fig. 3d). Importantly, no significant difference in TGI% was observed between Z15-0 and Z15-0-2, implying that the addition of Qα did not substantially affect the efficacy of the bispecific nanobodies.

### The introduction of Qα amplifies the expression level of Z15-0-2 mRNA

**Fig. 4:**
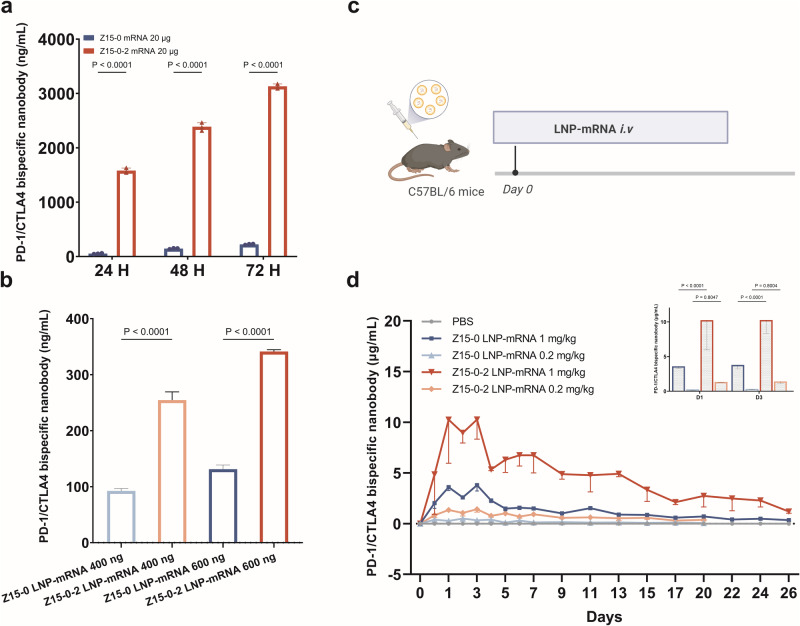
The insertion of Qα resulted in an elevation of Z15-0-2 mRNA expression both in vitro and in vivo. **a** CHO cells were subjected to electroporation, and the supernatant of the culture medium was collected at 24, 48, and 72 h. The secretion of nanobody was measured using ELISA. **b** CHO cells were transfected with LNP-mRNAs, and the supernatant of the culture medium was collected at 24 h to detect nanobody secretion using ELISA. **c** The administration schedule of LNP-mRNAs in C57BL/6 mice was followed. **d** ELISA was employed to assess the changes in antibody concentration in the serum of mice at various time points. All data were subjected to analysis using one-way ANOVA and showed as Mean ± SEM, *n* = 4.

To investigate the influence of Qα on mRNA expression levels, CHO cells (1.5E6) were electroporated with 20 μg of mRNA and subsequently cultured in a 3 mL medium. The supernatant from the culture medium was collected at 24, 48, and 72 h post electroporation, and the presence of bispecific antibodies was quantified using ELISA. As depicted in Fig. 4a, Z15-0-2 mRNA demonstrated significantly higher expression compared to Z15-0 mRNA at all time points, exhibiting an approximately 14-fold amplification at 72 h. These observations strongly suggest that the integration of Qα considerably augments mRNA expression levels.

Furthermore, CHO cells were transfected with varying doses of LNP-mRNAs to assess mRNA expression post-LNP transfection. As illustrated in Fig. 4b, Z15-0-2 exhibited a significantly higher secretion level of antibodies compared to Z15-0 at both 400 ng and 600 ng doses our findings unequivocally demonstrated that the expression level of Z15-0-2 significantly outperformed that of Z15-0 in vitro, as evidenced by electroporation and LNP transfection.

To evaluate the in vivo delivery of LNP-bispecific antibodies-mRNA, C57BL/6 mice were administered a single injection of Z15-0 or Z15-0-2 bispecific antibodies-mRNA at doses of 0, 0.2 or 1 mg/kg. Serum samples were collected and analyzed for the presence of bispecific antibodies following intravenous administration of LNP-mRNA as shown in Fig. 4d. No adverse events were observed and all doses were well tolerated by the mice. In the serum of mice, we observed a dose-dependent increase in levels of bispecific nanobodies on day 1 post-administration, reaching the peak on day 3 and rapidly declining by day 4. The in vivo expression findings for both bispecific antibodies were consistent with those obtained from in vitro experiments. Notably, during the peak expression period, the Z15-0-2 group exhibited significantly higher secretion of bispecific nanobodies compared to the Z15-0 group at an equivalent dosage, indicating that the inclusion of the Qα sequence significantly enhanced mRNA expression.

### Functional validation of two bispecific nanobodies LNP-mRNAs in vitro and in vivo

**Fig. 5:**
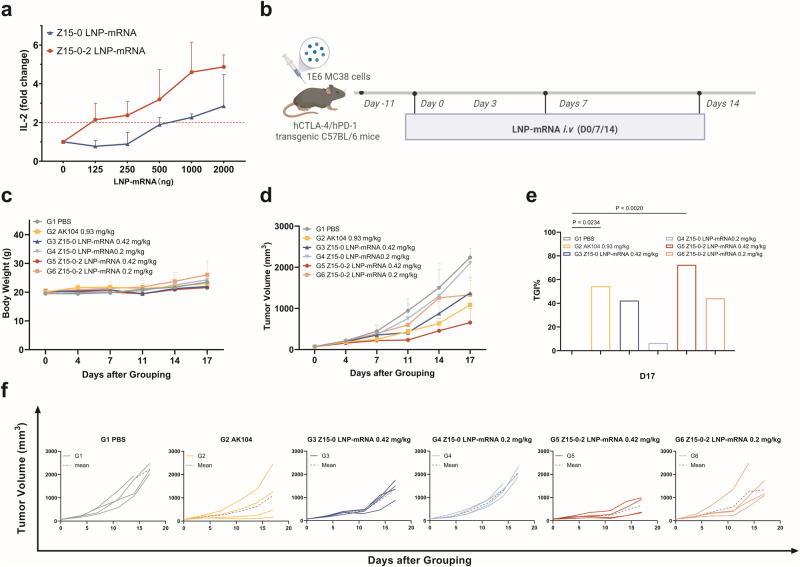
The evaluation of the efficacy of Z15-0 and Z15-0-2 LNP-mRNAs in vitro and in vivo. **a** The Mixed Lymphocyte Reaction (MLR) assay demonstrates that two bispecific nanobodies induce IL-2 secretion in a mixed culture of PBMCs and DCs. **b** illustrates the administration schedule of LNP-mRNAs hCTLA-4/hPD-1 in transgenic C57BL/6 mice. **c** Depicts the changes in body weight of the mice. **d** The representative tumor volume of the mice. **e** Compares the tumor growth inhibition (TGI) at D17 among different groups, with the G1-PBS group serving as the control. The data were analyzed using one-way ANOVA, *n* = 4 while (**f**) presents the individual tumor volume.

The functional and immunomodulatory activity of mRNA was evaluated using a mixed lymphocyte reaction (MLR), which involved isolating CD4 + T cells from human PBMCs and DCs obtained from a separate donor. As depicted in Fig. 5a, both Z15-0 and Z15-0-2 LNP-mRNA demonstrated a dose-dependent increase in IL-2 levels. Notably, Z15-0-2 LNP-mRNA exhibited a significantly higher secretion of IL-2 compared to Z15-0 LNP-mRNA, particularly at dosages of 1000 and 2000 ng. These findings collectively suggest that the utilization of Z15-0-2 LNP-mRNA holds promise for augmenting T-cell responses through enhanced IL-2 secretion.

To evaluate the efficacy of LNP-mRNA treatment in solid tumors, we established the same MC38 in vivo tumor model. The experimental design is illustrated in Fig. 5b, where AK104 was used as a positive control at a dosage of 0.93 mg/kg. Then, mice with MC38 tumors measuring an average size of 72.78 mm^3^ were given LNP-mRNA treatment. Throughout the experiment, the body weight of mice across all experimental groups remained relatively stable (Fig. 5c). Importantly, as shown in Fig. 5e, Z15-0-2 LNP-mRNA at a dose of 0.42 mg/kg exhibited higher TGI% (72.52%) compared to Z15-0 LNP-mRNA at the same dose (42.32%). This finding implies that the integration of Qα insertion can enhance antitumor efficacy by improving mRNA translation in mice. However, it should be noted that no significant tumor inhibition was observed in the low-dose group (0.2 mg/kg) treated with LNP-mRNA.

### Z15-0-2 actively remodels the TME

**Fig. 6:**
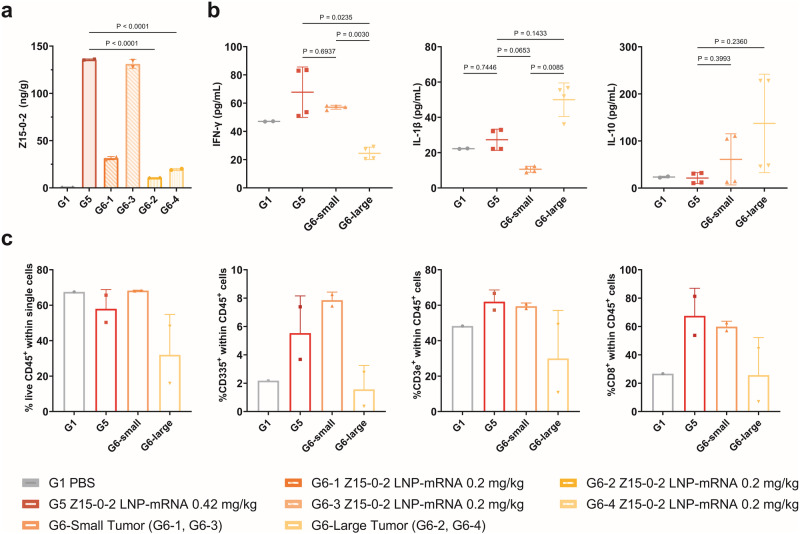
Z15-0-2 exerts its effect by remodeling the tumor microenvironment. **a** The content of Z15-0-2 in tumor tissues was detected by ELISA. **b** High-throughput protein liquid chip was used to detect the secretion of IFN-γ, IL-1β, and IL-10 in tumor microenvironment. All the data were analyzed using one-way ANOVA and showed as Mean ± SEM, *n* = 4**. c** Flow cytometry was used to detect lymphocyte population in tumor microenvironment.

To gain a deeper understanding of the enhanced efficacy of Z15-0-2 LNP-mRNAs in TME, we collected tumors from mice in both the PBS group and various dosage groups of Z15-0-2 LNP-mRNAs on day 17. These tumor tissues were utilized to measure antibody concentrations, cytokine concentrations, and immune cell clusters within the immune microenvironment, as depicted in Fig. 6.

To account for the variation in tumor volumes within the same group, we divided G6 mice into two subgroups based on tumor volume, a small tumor group (G6-1, G6-3) and a large tumor group (G6-2, G6-4). Afterward, we conducted an analysis to investigate the differences in their TME. The expression levels of Z15-0-2 bispecific nanobody showed variations across different tumor sizes, where antibody levels exhibited an inverse relationship with tumor size (Fig. 6a). Importantly, among mice with larger tumors, there was a significant increase in the expression of G5-0.42 mg/kg LNP-mRNA group compared to that observed in the G6-0.2 mg/kg LNP-mRNA group. Within the same group, mice with larger tumors showed significantly lower levels of G6-3 antibody compared to those with smaller tumors. Subsequently, we conducted a comparative analysis of the cytokine content in the tumors. As illustrated in Fig. 6b, the concentration of IFN-γ in the tumors of the G5 group was significantly higher than that of the G6-large group and similar to the G6-small tumor group. Additionally, both IL-1β and IL-10 concentrations were increased in the G6-large group when compared to the G5 group.

The TME is significantly influenced by immune cells [26]. Flow cytometry was used to assess the proportions of different subsets of immune cells in the TME. We showed that, both the G5 and G6-small groups showed an increase in lymphocytes, T cells, CD8+ cells, and natural killer (NK) cells compared to the G1-PBS control group. Within the G6 group, the proportion of immune cells also demonstrated a corresponding relationship with tumor size (Fig. 6c). These findings suggest that Z15-0-2 LNP-mRNA exerts antitumor effects by modulating the cytokine content and the composition of immune cell population in the TME.

## Discussion

In this study, we introduce a newly discovered bispecific nanobody, Z15-0, which has been specifically designed to simultaneously inhibit PD-1 and CTLA-4. Additionally, we have successfully optimized the mRNA sequence encoding this nanobody, known as Z15-0-2, to maximize its potential applications by utilizing efficient delivery methods such as LNP-mRNA technology.

AK104 and MEDI5752 are classified as IgG1 molecules, and Z15-0-2 falls into the IgG4 isotype due to the presence of stabilizing S228P Fc mutation. AK104 is designed with Fc null backbone that effectively inhibits the production of pro-inflammatory cytokines and immune effector functions. MEDI5752 incorporates modifications (L234F, L235E, and P331S) in the constant heavy chain of human g-1 to reduce ADCC activity [27]. Compared to IgG1, IgG4 exhibits lower binding affinity towards most Fcγ receptors, resulting in diminished ADCC [28, 29]. Additionally, Fab-arm exchange is hindered in vivo by the S228P mutation present in Z15-0-2 [30].

In contrast to MEDI5752, both AK104 and Z15-0-2 are tetravalent [5, 9]. Z15-0-2 is composed of two nanobodies targeting PD-1 and CTLA-4. These nanobodies possess dual binding sites and owing to the unique characteristics of nanobodies [31], Z15-0-2 may offer advantages such as small molecular weight, high stability, and low immunogenicity. In vivo experiments in mice demonstrated that while MEDI5752 could eliminate tumors in 60% of the mice when administered at a dose of 10 mg/kg, Z15-0-2 achieved TGI% of 90% at an injection dose of 0.5 mg/kg (Fig. 3d). MEDI5752 possesses the capability to facilitate the internalization and degradation of PD-1 by interacting with CTLA-4. Similarly, Z15-0-2 (400 nM) can facilitate approximately 20% of PD-1 endocytosis (measured at 4 h, data not published). Consequently, the structural design of Z15-0-2 exhibits potential for improved binding performance, which could potentially lead to enhanced antitumor effects.

The utilization of LNP-mRNA technology enables localized expression of antibodies or proteins, eliminating the need for time-consuming structural optimization procedures [32]. However, it is imperative to meticulously evaluate the in vivo efficiency of LNP-mRNA expression to ensure optimal drug performance. In this study, we improved the mRNA expression level both in vitro and in vivo by incorporating a sequence encoding Qα into mRNA. Previous research has demonstrated that Qα can augment protein expression and secretion by stabilizing mRNA [22]. Initially, we conducted a series of in vitro and in vivo experiments to provide evidence that the inclusion of Qα did not alter characteristics of the nanobody and the introduction of Qα did not compromise the protein ability to bind proteins or its antitumor efficacy. Figure 3d illustrates that there is no noticeable difference in TGI% between Z15-0 and Z15-0-2 achieving a TGI% comparable to that of AK104. Furthermore, we confirmed the increased mRNA expression level by performing in vitro electroporation with LNP transfection after integrating Qα. This finding was further supported by in vivo experiments conducted on mice (Fig. 4d), where Z15-0-2 LNP-mRNA exhibited significantly higher levels compared to Z15-0 LNP-mRNA at various delivery doses.

The effectiveness of bispecific antibodies in the TME is greatly influenced by their concentration. It is important to note that when administered through LNP-mRNA, Z15-0-2 shows a dose-dependent manner. Furthermore, we have observed a negative correlation between the abundance of bispecific antibodies within the TME and the magnitude of tumor size. In this study, we aimed to investigate the impact of Z15-0-2 on the TME as measured by the immune cells present.

Notably, the crucial roles of IFN-γ, IL-1, and IL-10 have been recognized in relation to tumor immunity [33]. Specifically, IFN-γ directly triggers apoptosis in tumor cells, boosting the presentation of antigens to immune cells, activating immune cells responsible for eliminating tumors, and suppressing cells that inhibit immune responses [34]. Although both IL-1 and IL-10 have the ability to promote cancer cell growth and support angiogenesis, leading to pro-cancer effects, it is worth noting that increased expression of IL-10 has been linked to the development of cancer and a negative prognosis in different types tumors [35]. Z15-0-2 in the TME was found to enhance higher levels of IFN-γ in the TME while significantly reducing the levels of IL-1 and IL-10 compared to G6-Large. Moreover, Z15-0-2 demonstrated its ability to regulate the distribution of lymphocytes, NK cells, and CD8+T cells in the TME. It is worth mentioning that the therapeutic effect of the antibody mainly depends on CD8+ T cells and NK cells [26, 36], suggesting its potential for reshaping the TME and exert anti-cancer effects.

In summary, we have successfully developed a unique bispecific nanobody, Z15-0, with the specific design to target PD-1 and CTLA-4 simultaneously. This antibody can be delivered either as a purified protein or as mRNA (Z15-0-2), which allows for efficient expression of the bispecific antibody. Notably, Z15-0-2 is compatible with the LNP-mRNA delivery system and has demonstrated significant efficacy in reducing tumor growth in a murine colon cancer model. Therefore, Z15-0-2 holds considerable potential as an innovative bispecific nanobody for tumor immunotherapy applications.

## Materials and methods

### Cell lines and culture conditions

The Chinese hamster ovary (CHO) cell line (ATCC, USA) was cultured in a 44% mixture consisting of Dulbecco’s modified Eagle’s medium (DMEM; Corning, 10-013-CVR) and Ham’s F-12K (GIBCO, 21127022). The culture media were further enriched with supplements including 10% fetal bovine serum (FBS; GIBCO, 10091148), 1% 200 mM L-Glutamine (GIBCO, 25030081), and 1% Hypoxanthine-Thymidine Supplement (HT Supplement; GIBCO, 11067030). For the cultivation of Jurkat-PD-1 (Shcell, China) cell line Roswell Park Memorial Institute (RPMI 1640; Corning,10-040-CM) supplemented with 10% FBS. Similarly, the 293T-CTLA-4 (Shcell, China) cell line was cultured in DMEM supplemented with the same amount of FBS. All tumor cells were detected by the PCR short tandem repeat (STR) method and screened for the presence of mycoplasma monthly. All these aforementioned cell lines were maintained with an incubator set to 37 °C under humidified conditions containing 5% CO_2_.

### Cells electroporation and transfection

In accordance with the guidelines provided by the Human T Cell Nucleofector™ Kit (LONZA, VVPA-1002), we obtained a total of 1E5 CHO cells during the logarithmic growth phase and resuspended in Human T Cell Nucleofector buffer with 20 μg of mRNAs. To ensure optimal transfer efficiency, we used the appropriate power transfer program CHO-K1 (H-014) Following established protocols, supernatant samples were subsequently collected at specific points (24, 48, and 72 h post electroporation) for subsequent analysis.

For in vitro transfection, 1.2 mL of medium was added to each well of a 12-well plate (NEST, 712001) followed by the addition of the prepared LNP-mRNAs. After a transfection period of 24 h, the cell culture supernatants were collected for subsequent ELISA analysis.

### LNP formulation and characterization

The LNPs were prepared following the procedure outlined in PCT/CN2022/118198. In summary, a lipid solution comprising an ionizable lipid, cholesterol, a phospholipid (DOPE), and DSPE-PEG200 (Shanghai Advanced Vehicle Technology L.T.D. Co) was prepared in ethanol. The molar composition employed was 35:46.5:16:2.5. To obtain an aqueous solution of mRNA, we diluted the mRNA in a 20 mM citrate buffer (pH = 6.1). Subsequently, liposomes were generated using a microfluidic device, by combining the ethanol lipid solution and mRNA solution at a volume ratio of 1:3 organic to aqueous phase, with a total weight ratio of approximately 10:1 for lipids to mRNA.

The LNP characterization was conducted using the Malvern Zetasizer Nano ZS (Malvern UK) with backscattering detection mode at a temperature of 173 °C. This method allowed for the measurement of the z-average diameter and polydispersity index (PDI) of the lipid nanoparticles through dynamic light scattering.

### Surface plasmon resonance (SPR) analysis

SPR analysis was employed to determine the binding avidity of the nanobodies with PD-1 and CTLA-4. According to the human antibody capture kit manual (cytiva, BR100839), the IgG (50 μg/mL) were immobilized onto a CM5 sensor. Subsequently, purified nanobodies were applied onto the pre-coated chip surface. Gradient concentrations (200, 100, 50, 25, 12.5, 6.25 nM) of the nanobodies were introduced to the chip surface at a flow rate of 30 μL/min to facilitate antigen capture by the IgG. Regeneration of the chip was carried out by employing a flow rate of 30 μL/min and a second 10 mM Glycine-HCl at pH 2.0 for a duration of 30 s. The data were collected by Fortebio Data Acquisition 7.0. Subsequently, Biacore Evaluation Software 2.0 was utilized to analyze the bond rate (kon), dissociation rate (koff), equilibrium constant (*K*_D_), and to fit the obtained curves using the Langmuir 1:1 model.

### High-throughput protein liquid chip

A high-throughput protein liquid chip, following the experimental techniques outlined by the PPX-06-MXZTFZG, was used to detect cytokines within the mouse tumor. The Luminex 200^TM^ System was used to quantify the mean fluorescence value (MFI), while a five-parameter nonlinear regression was applied to fit the standard curve. The obtained MFI values were then converted to their corresponding concentrations based on the established standard curve.

### Flow cytometry assay

Jurkat-PD-1 and 293T-CTLA-4 cell lines were incubated with bispecific nanobodies for 20 min at room temperature, followed by three washes with PBS. The washed cells were then stained with a MonoRab™ Rabbit Anti-Camelid VHH Cocktail conjugated with a phycoerythrin (PE) antibody (A02227, GenScript, China). The binding affinity of the bispecific nanobodies towards PD-1 and CTLA-4 on the cell surface was assessed using flow cytometry. Additionally, tumor microenvironment (TME) cells were surface-stained using fluorescently labeled antibodies targeting CD45(560510, BD Biosciences, USA), CD3e (557596, BD Biosciences, USA), CD8a (100762, Biolegend, California, USA), and CD335 (137611, Biolegend, California, USA). All samples were collected on Cytek (NC-CLC100, California, USA) or Beckman Coulter cytoFLEX (ONC-FCY-001, California, USA) and analyzed using Flow Jo software vX.0.7.

### Enzyme-linked immunosorbent assay (ELISA)

The expression of bispecific nanobodies in culture supernatants and sera from mice was performed using the ELISA. A 96-well microplate was coated with Human PD-1 (Acro, H5221) at 4 °C overnight. A concentration response curve was established by diluting Z15-0 and Z15-0-2 standard from 12.5 ng/mL down to a 2× dilution, resulting in 7 gradients and a concentration of 0 ng/mL. After cleaning and closing the plate, a MonoRab^TM^ Rabbit Anti-Camelid VHH Antibody (GenScript, A01861) was used as the detection antibody. The optical density (OD) values were measured at 450 nm using an EnVision system (PerkinElmer, Massachusetts, USA), and the curve of OD and antibody concentration was fitted using a Four Parameter Logistic (4PL) model.

### Mixed lymphocyte reaction (MLR)

Human peripheral blood mononuclear cells (PBMCs) were obtained from two healthy donors sourced from Shanghai Aoneng Biotechology Co., Ltd. (Shanghai, China) through Ficoll-Paque density gradient centrifugation. PBMCs from one donor were used for the preparation of CD4 + T cells, while the PBMCs from the other donor were used for DC induction. The specific procedure for DC induction can be found in CN107574149B. Additionally, CD4+ T cells were isolated from a separate donor’s PBMCs following the instructions provided by the manufacturer of the CD4+ T Cell Isolation Kit (Milteniy Biotech, Bergisch Gladbach, Germany).

During the experiment, mature dendritic cells (DCs) were washed with DPBS (Hyclone, SH30028.02) and then mixed with 2E5 mDCs in Opti-MEM™ (GIBCO, 31985070) medium within 24-well plates (NEST, 702001). The LNP-encapsulated mRNA was added at a concentration ranging from 0 to 2000 ng per well. The plate was then incubated at 37 °C with 5% CO_2_ for 4 h. Following this incubation period, 8E5 freshly isolated CD4+ T cells were added to each well and co-cultured for 5 days. After incubation, the culture supernatants were collected and assessed for the secretion of IL-2 using the Human IL-2 ELISA Kit (Sino Biological, KIT11848) following the provided manual instructions.

### In vivo experiments

All mice aged 6–8 weeks were obtained from PharmaLegacy (Shanghai, China) and housed in IVC Sealsafe cages under a 12/12 light/dark cycle at a temperature range of 20-26 °C. The animal-related experiments conducted in this study received approval from the Experimental Animal Ethics Committee of Shanghai University. Investigators were not blinded during data collection or analysis. Experimental and control animals were treated equally.

The C57BL/6 mice were used for pharmacokinetic evaluation, where LNP-bispecific nanobodies mRNA was intravenously (*i.v*.) administered at doses of 0.2 and 1 mg/kg through their tail veins. Blood samples were collected from the orbit and the serum was extracted for ELISA analysis.

The hCTLA-4/hPD-1 transgenic C57BL/6 mice were received subcutaneous injections of 1E6 MC38 tumor cells. Tumor volume (TV) was measured biweekly using calipers and the formula (width^2^ × length)/2. Once successful tumor engraftment occurred, with an average tumor volume of 72.78 mm^3^, the mice were randomly divided into groups receiving either administered subcutaneous LNP-mRNAs or bispecific nanobodies. To evaluate tumor growth inhibition (TGI), we employed a calculation method represented by (1 − *T*/*C*) × 100%, where *T* represents relative tumor volume in the experimental group compared to the control group at a specific time point denoted by *C*; meanwhile, *T*/*C* % indicates relative rate of tumor growth progression over time period examined. At the end of the experiment, all tumors were collected and weighed for further analysis purposes.

### Statistical analysis

Statistical analysis was conducted using GraphPad Prism software version 9.5.1. One-way analysis of variance (ANOVA), Student’s *t*-test and Tukey’s multiple comparison test was used to determine the statistical significance, with a *P*-value threshold of less than 0.05. The sample size for in vivo experiments was based on previous similar experiments in our laboratory. For all other studies, the data were replicated more than three times to ensure the robustness and reliability of the findings in line with rigorous scientific standards.

## Data Availability

The Z15-0 protein data in Fig. 1a and the Z15-0-2 mRNA data in Fig. 2a can be found in CN202310338674.1 and CN202311358648.1.
